# A Deep Learning-Based EIT System for Robust Gesture Recognition Under Confounding Factors

**DOI:** 10.3390/bios16040200

**Published:** 2026-04-01

**Authors:** Hancong Wu, Guanghong Huang, Wentao Wang, Yuan Wen

**Affiliations:** 1 School of Future Technology, South China University of Technology, Guangzhou 511442, China; 2 Pazhou Lab, Guangzhou 510320, China; 3 School of Natural and Computing Sciences, The University of Aberdeen, Aberdeen AB24 3UE, UK

**Keywords:** electrical impedance tomography, stretchable sensor, gesture recognition, deep learning, confounding factors

## Abstract

Gesture recognition with electrical impedance tomography (EIT) is an enormous potential tool for human–machine interaction because of its low cost, low complexity and high temporal resolution. Although high-precision EIT-based gesture recognition has been achieved in ideal scenarios, ensuring its consistent performance under interference remains challenging. This article presents a novel method to alleviate the effect of confounding factors on EIT gesture recognition. An EIT armband was designed to mitigate the effect of contact impedance variation based on equivalent circuit analysis, and a spatial–temporal fusion network, named the Fold Atrous Spatial Pyramid Pooling-Gated Recurrent Unit (FASPP-GRU), was developed for robust gesture classification. The results showed that the proposed two-layer electrode maintained a stable contact impedance when its contact force with the skin was changed. Although confounding factors caused significant changes in baseline forearm impedance, FASPP-GRU achieved 80% accuracy under the effect of limb position changes and dynamic changes in muscle state over time, which outperforms conventional classifiers. With an 87 μs inference time, the proposed system shows enormous potential in real-time applications.

## 1. Introduction

Gesture recognition is a type of human–machine interaction technique that captures hand movements or motor intentions and interprets their meanings. Leveraging the power of intelligent sensing, signal processing, and pattern recognition, gesture recognition has been widely employed in the control of bionic prostheses [1] or exoskeletons [2], neuromuscular diagnosis [3], neurorehabilitation [4] and virtual reality applications [5].

Conventional gesture recognition methods mainly include vision-based approaches [6,7], surface electromyography (sEMG) [8,9], and inertial measurement unit (IMU) [10,11]. Taking advantage of computer vision techniques, vision-based approaches can accurately recognize complex hand movements without attaching any sensors or devices to the users. However, these methods are constrained by spatial and environmental factors, including camera placement, ambient brightness and occlusion issues [12], and are not available for people with limb differences. Surface electromyography (sEMG) is a non-invasive technique for gesture recognition based on the monitoring of neuromuscular signals [1]. Due to its easy implementation and low resource requirement, sEMG has been widely applied in the control of commercial prostheses or exoskeletons [13]. However, since EMG signals are weak, the performance of these devices in daily living conditions is degraded by measuring noise and confounding factors [14]. Similarly, IMU relies on tracking limb movements and is highly sensitive to changes in sensor placement and orientation. Its accuracy can diminish in unconstrained activities where gestures lack a fixed starting posture or are performed with variable speed. Hence, the exploration and development of novel sensing modalities for robust and reliable gesture recognition have attracted extensive research attention.

Electrical impedance tomography (EIT) is a non-invasive technique for human body monitoring. By measuring the corresponding boundary voltage for predefined injected current patterns, EIT reconstructs the conductivity distribution within the sensing area [15] for functional analysis. With the advantages of being low-cost, non-invasive and having a high temporal resolution, EIT has been applied in applications of chronic obstructive pulmonary disease (COPD) management [16] and brain injury detection [17]. Furthermore, it has also been demonstrated to be an effective gesture recognition approach in recent years. Wu et al. designed an EIT system to predict hand movements by monitoring forearm muscle activities [18]. The system was deployed with an eight-electrode armband and the adjacent current drive pattern for measurement, which successfully applied EIT in prosthetic control for the first time [19]. Yao et al. demonstrated the effect of electrode–skin contact impedance on classification accuracy by comparing different materials and shapes of the electrode [20]. Liebing et al. designed an armband with 32 electrodes and compared the performance between two-pole and four-pole measurement schemes [21]. The results showed that the four-pole measurement scheme was more robust regarding noise. Compared with conventional machine learning algorithms, such as decision tree [22], Support Vector Machine (SVM) [23,24] and K-Nearest Neighbor (KNN) [25], deep learning models like Convolutional Neural Networks (CNNs) [26] and ResNet with Bayesian optimizer [27] show a better performance when the number of gestures increases. Wang et al. proposed an improved neural network, PEO-SFU-ResNet50, to enhance the classification robustness of the model [28]. However, existing studies are mostly conducted under ideal conditions, with limited consideration of the influence of confounding factors in real-world applications [26,29]. To address this issue, our work presents two key novelties: First, we consider the effect of sensor configuration on measurement results and design an electrode that is less susceptible to interference. Second, in terms of algorithm design, we further evaluate common disturbances in practical gesture recognition scenarios and develop a more robust algorithm accordingly.

This study investigates the impact of confounding factors on EIT signals during hand motions and proposes a novel EIT gesture recognition system that mitigates their effects on the accuracy of gesture recognition. The main contributions of this study are described as follows:

(1) A 16-channel stretchable EIT armband is developed for the acquisition of data for the forearm. The armband is made of elastic materials so that it can fit different participants. A novel structure is adopted in the design of the electrode, which maintains a steady electrode–skin interface to provide stable boundary voltage measurements.
(2) A protocol is designed to develop an EIT dataset for gesture recognition under real-life settings. Three confounding factors for gesture recognition in daily life, including limb position interference, time interference and contact interference, are investigated. Both transient-state and steady-state data are collected, which provides additional information for model training.
(3) A gesture recognition model, named FASPP-GRU, is proposed for high-accuracy gesture recognition under confounding factors. Spatial–temporal features from EIT measurements are extracted with the integration of an Atrous Spatial Pyramid Pooling (ASPP) module. Fold operation is applied to enhance the spatial correlation between sampling points and, thus, generates more stable features.

## 2. Methodology

### 2.1. Design of Stretchable EIT Armband

**Figure 1:**
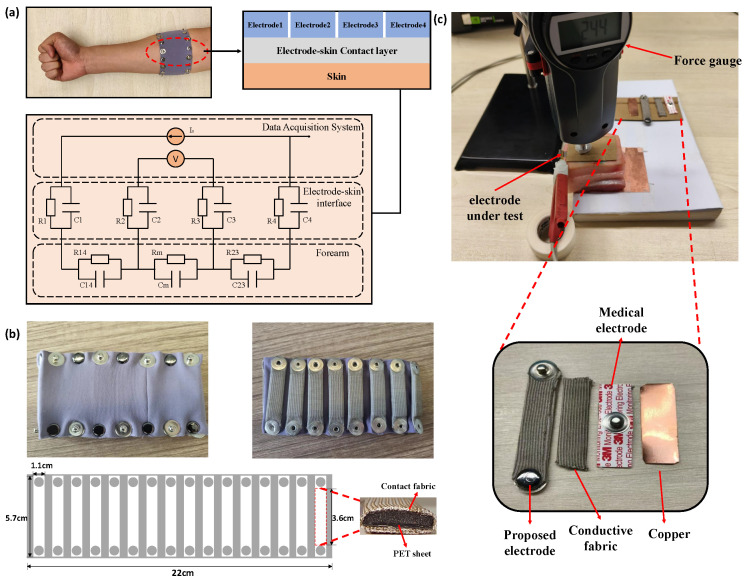
Design and evaluation of the stretchable EIT armband. (**a**) The equivalent measuring circuit. (**b**) Structure of 16-channel EIT armband. (**c**) Comparison experiment schematic diagram.

The principle of EIT-based gesture recognition is to detect biological impedance changes during hand motions and map them to distinct gestures. The equivalent measuring circuit is shown in Figure 1a, where R1//C1 to R4//C4 indicates the contact impedance between four electrodes and the skin and ‘//’ denotes that two components are connected in parallel; R14//C14 and R23//C23 are the edge impedance between electrodes 1–4 and 2–3; and Rm//Cm is the equivalent impedance of the region of interest (ROI). To mitigate the interference caused by contact impedance and edge impedance, electrodes should be maintained at the same positions and variance in contact impedance needs to be eliminated during the measurement.

To achieve the above requirements, we designed a 16-channel EIT armband as illustrated in Figure 1b. The armband had a circumference and width of 22 cm and 7 cm, respectively. Electrodes were evenly spaced on the armband, with each electrode measuring 6 cm in length and 1.1 cm in width. A two-layer electrode structure was designed, which covered a PET film with silver fiber. The silver fiber increased friction between the electrodes and the skin, thereby preventing displacement during arm movement, and the PET film was designed to enhance the toughness of the electrode.

As can be observed in Figure 1a, the impedance measured by the EIT system was composed of the boundary impedances R2//C2, R3//C3 and the impedance of the region of interest (ROI) Rm//Cm. To obtain accurate impedance changes in the ROI, we needed to mitigate changes in boundary impedance due to pressure changes, which may be caused by muscle contraction during gesture execution. As shown in Figure 1c, a comparative experiment was conducted to verify the stability of contact impedance under different pressures. A piece of streaky pork was placed on the experimental desk and a copper sheet was placed beneath it to be the negative electrode of the testing circuit. Copper, conductive fabric, a 3M medical electrode and the proposed electrode were placed on the surface of the streaky pork to be the positive electrode, respectively. A force gauge was held on the top of the electrode under test to apply pressure from 0 to 10 N, with a 2 N increment, and the impedance between the electrode and the copper sheet was probed by ISX-3 impedance analyzer (Sciospec Scientific Instruments GmbH, Bennewitz, Germany) with a 100 kHz stimulation frequency. Impedance variations between different pressures were analyzed and compared.

### 2.2. Design of EIT-Based Gesture Recognition System

**Figure 2:**
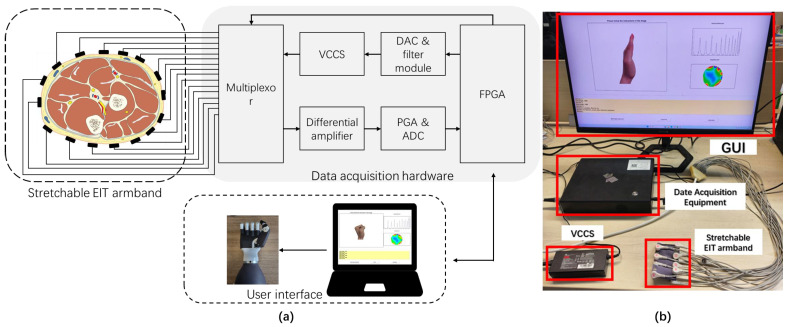
The (**a**) architecture and (**b**) photo of the EIT gesture recognition system.

An EIT system was designed for the gesture recognition study, which gave the command to the participant and monitored the morphological changes of forearm muscles through EIT measurement. The architecture diagram of the system is shown in Figure 2a. A V100 EIT system (V100 EIT system, YuanLu Inc., Shenzhen, China) was used for data acquisition, which measured the EIT data at 100 kHz with a speed of 23 frames per second. A customized graphical user interface (GUI) was designed using PyQt 5.15.10 (Python bindings for the Qt application framework, Riverbank Computing Limited, Dublin, Ireland), as shown in Figure 2b, for the interaction between the participant and the EIT hardware. It included a control panel to set the data acquisition hardware, a visualization module to show the boundary EIT measurement and the conductivity image in real time, and a command window for giving instructions to the participant. During the experiment, participants followed the instructions on the command window to grasp their hand, and the corresponding EIT measurements were collected and labeled by the system. After data collection, the train button on the control panel allowed the system to train the gesture recognition algorithm described in the following sections, which could be applied in human–machine interactions, such as prosthetic control.

### 2.3. EIT Image Reconstruction

The principle of EIT is to estimate the conductivity distribution within the sensing area based on the boundary current injections and corresponding voltage measurements [15]. The linearized EIT image reconstruction model is given by:

(1) ΔV=JΔσ

where *J* denotes the sensitivity matrix and ΔV and Δσ are the boundary voltage and internal conductivity, respectively.

Since solving Equation (1) is an ill-posed inverse problem, Tikhonov regularization is applied to confine the solution space for getting a unique stable solution:

(2) $$\Delta \sigma ={\left(J^{T}J+\alpha I\right)}^{-1}J^{T}\Delta V$$

where *I* is an identical matrix and α is the regularization parameter.

In this study, image reconstruction is only used to demonstrate the conductivity distribution of the forearm. Gesture recognition is based on the gesture classifier, which purely relies on the boundary voltage measurements.

### 2.4. FASPP-GRU Robust Gesture Classifier

**Figure 3:**
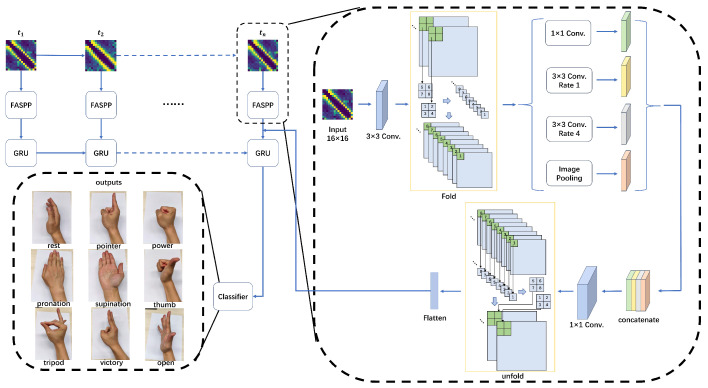
Structure of the proposed network.

Figure 3 shows the architecture of the proposed confounding-factor-tolerant network, called the Fold Atrous Spatial Pyramid Pooling-Gated Recurrent Unit (FASPP-GRU). Considering that reference voltages for image reconstruction are not always available in gesture recognition scenarios, we use raw EIT data as the input. The input layer contains an EIT measurement sequence lasting for 480 ms, where each frame is reshaped into a 16 × 16 matrix [26]. First, the matrix is upsampled to obtain a high-resolution feature map, which is then processed by the “fold” operation. The “fold” operation is a tensor transformation operation that uses a 2 × 2 sliding window to extract local blocks from the input feature map and separate them into new vectors while preserving the relative positions. In this process, the height and width of the feature map are reduced while the channel dimension is increased [30]. Next, multi-scale atrous convolutions are performed on the folded feature map to capture spatial features of the feature map and expand the receptive field without losing spatial resolution. After restoring the EIT measurement space using the “unfold” operation, an operation inverse to the folding subdivision, a GRU is employed to capture the temporal dependencies of the input sequence. It aims to analyze how the spatial conductivity features at each moment evolve and correlate with previous states. Finally, the extracted features are classified using a multi-layer perception, with the cross-entropy loss defined in Equation (3).

(3) $$L=-\sum_{i=1}^{C}y_{i}\log\left({\hat{y}}_{i}\right)$$

where *C* denotes the number of categories and $y_{i}$ and ${\hat{y}}_{i}$ represent the one-hot encoding of the ground truth, which is labeled in Section 2.5, and the predicted probability of the model, respectively.

### 2.5. Data Collection Protocol

Eight able-bodied participants took part in this study. The experiment was approved by the ethical committee of the The Second Affiliated Hospital of South China University of Technology (approval number: S202310801). All participants provided informed consent according to the Declaration of Helsinki before participating in the study.

The purpose of this study was to investigate the effect of confounding factors on gesture recognition based on EIT and the capability of different algorithms to mitigate these effects. Therefore, the experiment was designed to collect EIT signals corresponding to various gestures when the muscles were affected by different interferences.

**Figure 4:**
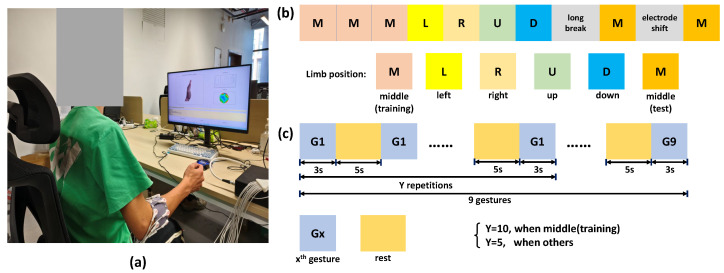
Experimental protocol with (**a**) participant sat in front of the GUI and following (**b**) the program to collect gesture data at different limb positions, and (**c**) data collection protocol for each limb position block.

Participants sat in an experimental chair with the right palm facing inward towards the body (Figure 4a). An LCD screen was placed about 0.5 m in front of the participant to show the GUI, which gave instructions during the experiment. The proposed EIT armband was worn on the participant’s right forearm, with the first electrode placed on the surface of the extensor carpi radialis (ECR), a group of forearm muscles which act as the primary wrist extensors and radial deviators on the posterolateral aspect of the forearm. Measurements were taken with 100 kHz stimulation frequency using the adjacent current drive method, where currents were injected into the forearm through two neighboring electrodes and voltages were measured across all other adjacent electrode pairs. Data visualization was performed before the data collection to ensure all electrodes were correctly connected.

The experimental protocol is presented in Figure 4b, where EIT data for a total of 9 commonly used human gestures were collected in this study, including pointer, power, pronation, supination, thumb up, tripod, victory, open and rest. The whole experiment was divided into two parts. The first part collected EIT data under no interference for model training, which included three sessions, as indicated by the pink grid cell marked with M in Figure 4b. In each session, participants started with the hand at rest and followed the instructions on the GUI to make each gesture, respectively. Each gesture instruction was repeated 10 times per session. EIT data were collected within the 3 s instructed period, followed by a 5 s interval. The second part was divided into three stages. The first stage, labeled L, R, U, and D in Figure 4b, collected EIT data for the above gestures when the limb position of the participant was changed to the left, right, up, and down positions, respectively. The second stage required participants to wear the EIT armband for over two hours to mimic the interferences caused by long-term usage of the system, such as variations in body temperature and skin impedance, and then another experimental block was conducted, indicated by the orange grid cell marked with M in Figure 4b. Finally, participants rotated the armband anticlockwise for the width of half an electrode to collect the data under contact inference in the last orange grid cell marked with M. For all experimental blocks in the second part, each gesture was repeated 5 times with a 3 s duration, and the interval between two repetitions was 5 s.

To construct a dataset for model training and testing, we integrated and labeled the EIT data collected through the above experiment. Data generated during the 3 s instructed period were labeled according to gestures shown on the screen, while all data with the same label were combined to form the dataset for the corresponding gesture.

### 2.6. Conventional Classifiers for EIT Gesture Recognition

Three well established methods were applied for comparison: K-Nearest Neighbors (KNN) [31], Support Vector Machine (SVM) [32], and CNN-LSTM [33]. Both KNN and SVM are classical machine learning algorithms. KNN determines the class by finding the nearest neighbors for voting, while SVM focuses on finding the optimal decision boundary that most clearly and widely separates different classes. They have been commonly used in previous EIT-based gesture recognition studies. CNN-LSTM is a typical spatiotemporal feature extraction model that combines the powerful feature extraction capability of CNN with LSTM’s strength in fully extracting time-series features, making it suitable for processing data with temporal and spatial dependencies.

### 2.7. Model Training

All models were constructed and trained on the computer with an NVIDIA RTX 4060 Ti and an Intel i7-13700F Processor. To train the model, the data in three training blocks were segmented into sequences with a length of 480 ms and an overlapping rate of 87.5%. A randomized 70-15-15 training–validation–test split was applied. Adam optimizer was employed to optimize the model parameters with an initial learning rate of 5 × 10^−5^. We plotted the learning curves of the loss and accuracy on the training set and validation set to analyze the convergence of the model. The stopping criterion was determined by both the loss and the maximum iteration. For machine learning-based methods, hyperparameters were fine-tuned to optimize the performance of each model.

### 2.8. Evaluation Matrices

We evaluate the performance of the above models using accuracy and *F*1-score. consistent with the equation. Please confirm. The equations are defined as follows:

(4) $$Accuracy=\frac{TP+TN}{TP+TN+FP+FN}$$

(5) $$F1=2\times \frac{Precision\times Recall}{Precision+Recall}$$

where TP, TN, FP and FN are the number of true positives, the number of true negatives, the number of false positives and the number of false negatives, respectively. Precision is defined as TP/(TP+FP), while Recall is defined as TP/(TP+FN).

## 3. Results

### 3.1. Evaluation of the Electrode Performance

Table 1 demonstrates the changes in contact impedance caused by the external pressure. With an increase in applied pressure, a significant drop can be observed from the copper electrode and the conductive fabric electrode, which is mainly due to the change in contact impedance. The 3M electrode provided the most stable measurements under varying contact pressures, but it is unsuitable for wearable armband applications due to its reliance on conductive gel. Compared with the copper and conductive fabric, the proposed electrode design significantly reduces the variations in contact impedance between different pressures, which ensures the consistency of EIT measurement during human activities such as muscle contraction.

### 3.2. Analysis of the Impedance Pattern

**Figure 5:**
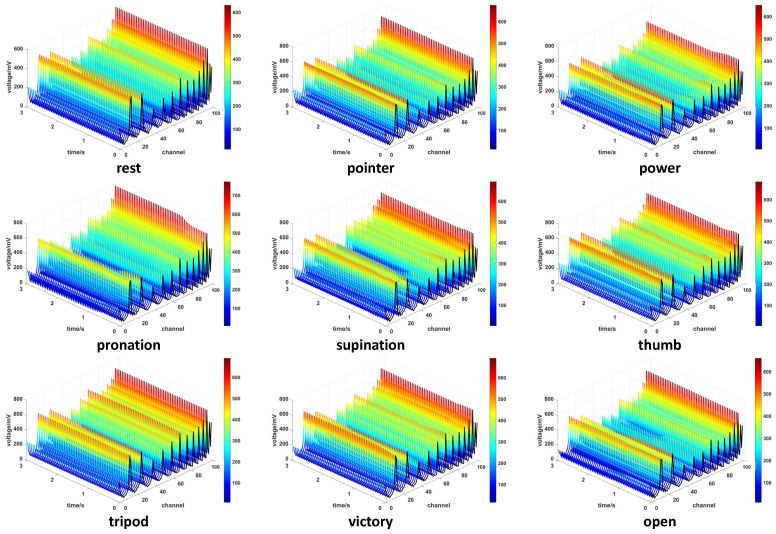
Samples of EIT measurements for each gesture. Recording was started before the hand movement in order to monitor effects of transient finger movements to the boundary voltages.

Figure 5 shows examples of EIT patterns for the nine gestures. With the baseline voltages varying between 20 and 800 mV, different gestures cause distinct voltage responses. For example, the boundary voltage in channel 99 increases by 1.5% when the participant straightens their thumb and bends the remaining fingers to make a “thumb” gesture, while it reduces by 10.7% for pronation. While similar voltage patterns can be observed between “power” and “thumb” because the movements of four fingers between these two gestures are the same, the transient states between them are different due to the order of finger movements. The transient state from rest to a gesture varies from 0.4 s to 1.2 s, depending on the participant and the complexity of the hand movement. Therefore, capturing the muscle activities in detail with high temporal–spatial resolution by the proposed system is helpful for training accurate gesture recognition models.

### 3.3. Visualization of the Muscle Activities

**Figure 6:**
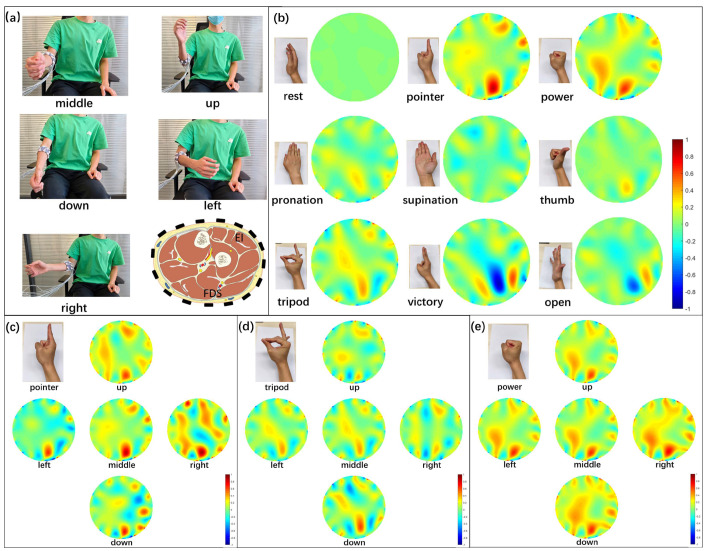
Reconstructed image samples corresponding to gestures and limb positions. (**a**) Demonstration of the limb position and the forearm muscle distribution, (**b**) samples of the reconstructed images for nine gestures at the middle limb position, and (**c**–**e**) reconstructed images for pointer, tripod and power at five limb positions, respectively.

To analyze the impact of limb position changes, cross-sectional images for forearm activities are reconstructed in Figure 6. Changing the hand from one gesture to another requires the contraction of the forearm muscles, which leads to changes in muscle hardness and small morphological alterations in other tissues. For example, changing the hand from relaxed to pointer needs the extension of the index finger and the flexion of the middle, ring and little fingers, which corresponds to the activation of the extensor indicis (EI) and flexor digitorum superficialis (FDS) (Figure 6a). Corresponding conductivity changes are observed in the reconstructed image labeled “pointer” in Figure 6b, where boundary voltages for the "rest" gesture are used as the reference. Morphological alterations in other muscles and tissues are reflected in mild conductivity changes in the background area.

Figure 6c–e show the effects of limb position changes on reconstructed EIT images for selected gestures. For the “pointer” gestures, changing the limb position from middle to up, right, and down causes obvious perturbations in the background of the EIT images. Similar changes are observed in the images for the “tripod” gesture, but the influence of limb position changes on the images for the “power” gesture is significantly smaller. One reason is that the flexor digitorum superficialis, flexor digitorum profundus, flexor carpi radialis, and flexor pollicis longus muscles are contracted strongly to hold the “power” gesture, and the muscle activities for changing limb positions are overwhelmed because those muscles are close to each other.

### 3.4. Gesture Recognition Results

#### 3.4.1. Overall Performance

**Figure 7:**
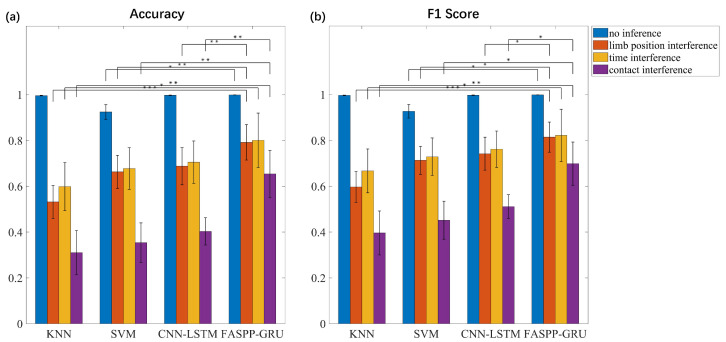
Classification accuracy and F1-score of the EIT dataset under different confounding factors. Error bars indicate the variance of the results between participants. Friedman test is used to compare the performance of FASPP-GRU with other methods, significant differences are makred as: * *p* < 0.05, ** *p* < 0.01 and *** *p* < 0.001.

To evaluate the performance of our EIT gesture recognition system, we analyzed the classification accuracy under different conditions. Figure 7 compares the overall classification accuracy and F1-scores between KNN, SVM, CNN-LSTM and the proposed model under an ideal scenario and three confounding factors. It can be observed that the four models show good performance when no inference occurs, achieving over 92% accuracy in the nine-class classification task. Among the three confounding factors, the contact inference has the most significant effect on the classification accuracy, which results in a 60% accuracy drop for KNN and 47.3% for SVM, respectively. Among all participants, the gesture recognition accuracy under time inference has the largest variance for all the models, which may be due to different activities during the long break. Compared with KNN, SVM and CNN-LSTM, the proposed method shows significant improvement in both the accuracy and F1 score for results under three confounding factors, especially under the contact inference. Although FASPP-GRU shares a similar performance with CNN-LSTM when no inference occurs, its average classification accuracy is 10.4%, 9.5% and 25.1% higher than CNN-LSTM under limb position inference, time inference and contact inference, respectively.

#### 3.4.2. Confusion Matrix

**Figure 8:**
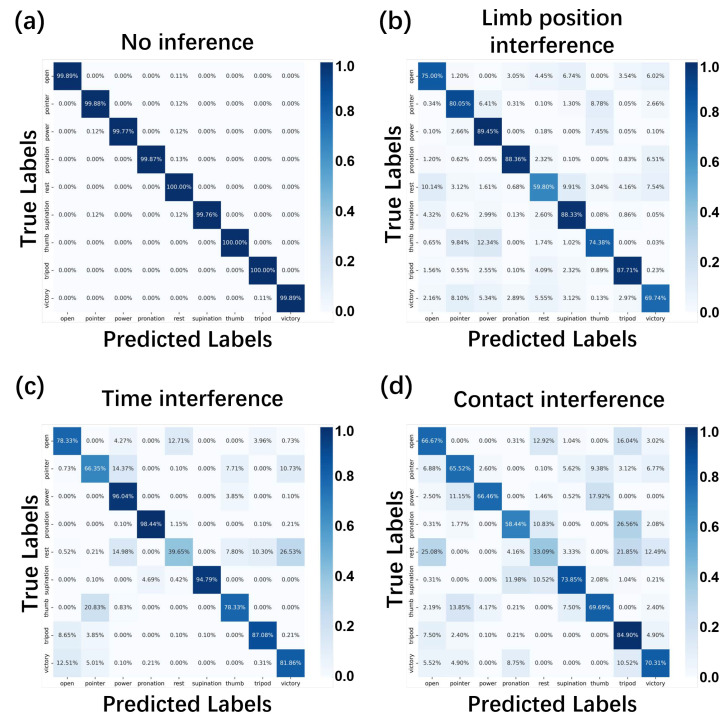
Confusion matrices of the FASPP-GRU model under different interferences.

Figure 8 shows the confusion matrices of the FASPP-GRU model for four circumstances. We collect all the testing data for the eight participants under the same type of interference to comprise the test dataset; hence, the number of samples in each class is balanced. The classification accuracy is 99.76% or higher for every gesture when no interference exists (Figure 8a). Significant misclassifications occur in similar gestures among three different types of interference (Figure 8b–d), especially when the hand is at rest. One reason is that confounding factors change the conductivity distributions of the forearm and the electrode–skin surface, which offsets the boundary voltages to patterns similar to a hand gesture. The “pointer” gesture is easily recognized as the “thumb” or “victory” gesture under confounding factors because of similar finger behaviors (i.e., naturally close the ring and little fingers). For the similar reasons, “thumb” and “open” have relatively higher error rates under limb position interference and time interference. Gestures including “power”, “pronation”, “supination” and “tripod” maintain high accuracy when limb position interference and time interference exist because they have unique muscle contraction strategies.

#### 3.4.3. Analysis of the Limb Position Effect on Gesture Recognition

**Figure 9:**
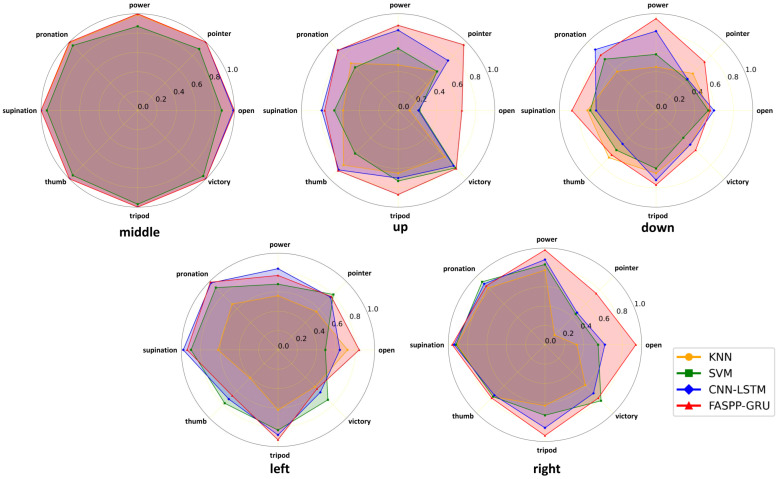
Average gesture recognition accuracy at different limb positions.

The effect of limb position changes on gesture recognition is demonstrated in Figure 9 in detail, which shows the average results among the participants. When all the gestures reach over 80% accuracy at the middle limb position, significant accuracy drops can be observed for KNN, SVM and CNN-LSTM. For these three models, moving the forearm up, down and right reduces the accuracy for at least one gesture to below 50%, which degrades the stability of the system when using it in real-life scenarios. On the other hand, FASPP-GRU maintains an accuracy over 80% for most of the gestures, and none of them go below 55% after limb position changes. For the recognition of frequently used gestures like power and open, FASPP-GRU greatly outperforms the other three models in four limb positions, especially at the right limb position, which reaches accuracies of 98% and 94%, respectively.

**Figure 10:**
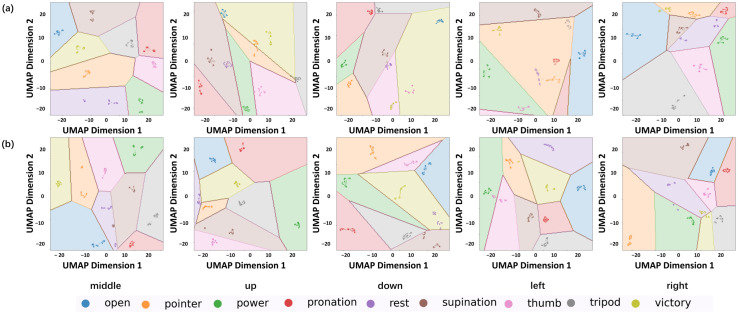
UMAP visualization of a subject at different limb position using (**a**) CNN-LSTM and (**b**) FASPP-GRU.

To further analyze the model behavior, Uniform Manifold Approximation Projection (UMAP) was used to reduce the feature space from high to two dimensions. UMAP is a powerful nonlinear dimensional reduction method based on Riemannian geometry and topological data analysis, which effectively preserves both local and global structures of high-dimensional data when projecting it into low-dimensional spaces. As depicted in Figure 10, UMAPs are plotted for CNN-LSTM and FASPP-GRU under five limb positions. The color of overlapping clusters indicates the true label for the feature points, while the color blocks in the background indicate the decision areas for different gestures by the classifier. Although both methods show good UMAP features in the middle limb position, limb position changes cause significant domain shifts for CNN-LSTM. For the four limb position changes in Figure 10a, clusters tend to move into their adjacent decision areas, and some decision areas vanish in the figure. For example, pronation, rest, tripod and victory are classified as pointer by CNN-LSTM after moving the limb left. In contrast, FASPP-GRU keeps the clusters at the centers of the correct decision areas, showing more robustness to limb position changes (Figure 10b).

#### 3.4.4. Performance Analysis

**Figure 11:**
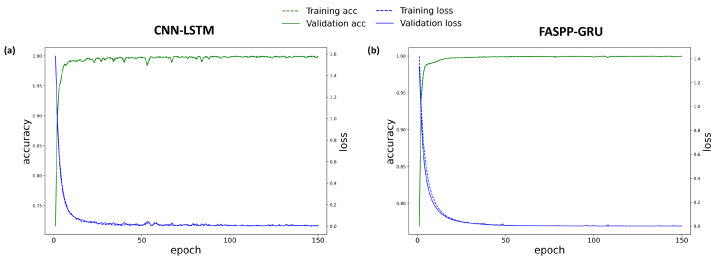
Average learning curve for the middle limb position training data: (**a**) FASPP-GRU, (**b**) CNN-LSTM.

Figure 11 depicts the average learning curve over eight participants. While CNN-LSTM requires 100 epochs to converge (Figure 11a), we observe that both training and validation accuracy and loss for FASPP-GRU converge to a plateau after 60 epochs (Figure 11b). This indicates that the model is able to learn the full data distribution. The FASPP-GRU model has 0.65M parameters, which gives it a lower complexity compared with the CNN-LSTM model, whose parameter size is 0.88M. The average inference time of the FASPP-GRU model is 87μs per sample (11.5 frames per second), which satisfies the requirement for real-time implementation.

## 4. Discussions

Confounding factors are one of the most significant barriers for EIT-based gesture recognition to be applied in real-life scenarios [34]. To overcome this barrier, we analyzed the reason for the degradation of gesture recognition systems under interference conditions and, therefore, designed a novel system to mitigate the effect of confounding factors.

Conventional 16-channel four-point electrical impedance tomography (EIT) was adopted as the data acquisition method in this study, yielding 208 independent measurements to enable accurate identification of complex gestures by capturing subtle impedance changes induced by muscle activity. To mitigate interference from contact impedance variations, we proposed a novel electrode design to reduce contact impedance discrepancies under different contact pressures, ensuring measurements more reliably reflected muscle-movement-related impedance changes. Such a design improved the system performance under limb position interferences and time interferences, but had limited impact on contact interference due to contact impedance drift. The application of extra voltage measurements at current injection electrodes may help the detection of drifting contact impedance, but it has not yet been applied in this work. Single-frequency EIT captures the lumped impedance of both the electrode–skin interface and forearm tissues, making it difficult to decouple contact resistance drift from authentic gesture-related signals. Integrating these extra measurements would only introduce severe interference noise.

To overcome the limitations in hardware design, we proposed a novel gesture recognition model, named FASPP-GRU, using a deep learning approach. The proposed FASPP-GRU classifier exhibits outstanding robustness for EIT-based gesture recognition under confounding factors, which outperforms conventional KNN, SVM and CNN-LSTM models remarkably. The fold–unfold atrous spatial pyramid pooling module effectively captures multi-scale spatial features without resolution loss, while the GRU layer accurately mines the temporal dependencies of dynamic EIT sequences. This architecture addresses the accuracy degradation caused by confounding factors, especially the severe drop induced by limb position changes. It provides a reliable and interference-tolerant solution for practical EIT gesture recognition applications.

## 5. Conclusions

In this paper, we propose a novel gesture recognition system using an EIT sensor and deep learning-based classifier. A stretchable EIT armband was designed to maintain a stable electrode–skin contact interface, and a multi-scale spatial–temporal convolutional network, named FASPP-GRU, was developed using both the transient-state and steady-state gesture data. Compared with existing studies, we investigated the most common confounding factors for gesture recognition independently and mitigated their impacts from the perspective of both sensor design and classifier innovation. Three confounding factors, including limb position interference, time interference and contact interference, were applied to evaluate the robustness of the system. Although experimental results show that confounding factors cause significant drift on the forearm conductivity distribution, the proposed method maintains high gesture recognition accuracy and outperforms conventional approaches. This indicates that our proposed system has the potential to be applied in real-life scenarios. As a preliminary study, experimental data in our research were collected under strict constraints, where only one kind of confounding factor was applied at a time. Future studies will focus on the investigation of EIT gesture recognition under more complex circumstances.

## Figures and Tables

**Table 1:** The result of impedance analysis experiment.

Electrode Material	Contact Area/mm^2^	Pressure and Impedance (Ω)					
		0 N	2 N	4 N	6 N	8 N	10 N
Copper	36 × 11	2404 ± 16	2118 ± 15	2007 ± 10	1925 ± 12	1852 ± 10	1779 ± 11
3M electrode	36 × 11	957 ± 14	912 ± 14	913 ± 16	898 ± 12	893 ± 10	897 ± 14
Conductive fabric	36 × 11	802 ± 10	508 ± 5	466 ± 5	444 ± 3	429 ± 3	414 ± 2
Proposed electrode	36 × 11	615 ± 8	520 ± 3	495 ± 3	483 ± 5	471 ± 4	461 ± 3

## Data Availability

The raw data supporting the conclusions of this article will be made available by the authors on request.
